# Integration of scientific research training into undergraduate medical education: a reminder call

**DOI:** 10.3402/meo.v18i0.22832

**Published:** 2013-10-21

**Authors:** Ahmed Abu-Zaid, Khaled Alkattan

**Affiliations:** College of Medicine, Alfaisal University, Riyadh, Saudi Arabia

**Keywords:** scientific research, undergraduate, medical, education, evidence-based medicine, translational research

## Abstract

There is an increasingly growing trend towards integrating scientific research training into undergraduate medical education. The importance and compulsoriness of this trend has been greatly highlighted at the Boyer Commission on Educating Undergraduates in the Research University. Despite the importance and benefits of undergraduate research, attempts of medical schools to encourage undergraduates to take part in formal research training during undergraduate medical education remain unsatisfactory. This article serves as a ‘reminder call’ highlighting the requisite to integrate scientific research training into undergraduate medical curricula.

There is an increasingly growing trend towards integrating scientific research training into undergraduate medical education. The importance and compulsoriness of this trend has been greatly highlighted at the Boyer commission on educating undergraduates in the research university (1).

The Boyer commission report has dramatically reformed how universities should approach undergraduate education. In particular, it was advocated that research-based learning should be the standard for any given undergraduate education. Since then, the integration of teaching and scientific research into undergraduate medical curricula has been greatly emphasized and employed in Western countries (1).

Medical schools have different methods of engaging its students in scholarly undergraduate research activities. These methods include research-driven curricula (1), research electives (2), compulsory research projects for graduation (3), and others.

Exposure to scientific research promotes scientific output (4) encourages interest in prospective research activities (5) and ultimately facilitates access to physician–scientist (research-focused) professions (4). Furthermore, it allows students to build up their scientific research knowledge, master basic laboratory skills, develop higher order research competencies such as critical-thinking, problem-solving, thought-processing, wise-judgeing, life-long learning, hypothesis formulation, methodology delineation, results interpretation and data communication both orally and textually (2, 3, 6). It is indisputable that such concrete scientific research competencies are of clinical relevance to patient care regardless of the healthcare/medical specialty. In addition, at the present time, research expertise is a highly desired (if not requisite) competency for every well-trained medical practitioner.

Little is known about how medical students perceive undergraduate research (7). Students’ development of positive attitudes towards scientific research is an essential component of modern undergraduate medical education (8). Broadly speaking, attitudes towards scientific research and a future research profession are considerably influenced by preceding training in scientific research (9), encouraging faculty staff (10), incentive research-supporting environment (11), gender differences (12, 13), institutional hindrances, and cultural obstacles. In addition, there are limited studies that attempted to explore barriers to undergraduate research among medical students (10, 14). Such barriers included overwhelming academic workload (10), lack of time to pursue scientific research activities (10), and insufficient, thorough instruction of the basics of scientific research at medical colleges (15).

At present, the practice of medicine is largely dependent on utilization of the contemporary evidence-based medicine. Evidence-based medicine is defined as ‘the conscientious, explicit and judicious use of current best evidence in making decisions about the care of individual patients’ (16). As evidences are largely based on research studies and critical interpretation of their findings, it is necessary for prospective healthcare providers to be well-familiarized with scientific research so that they can properly interpret research results and accordingly opt for the best care decisions for their patients. In the settings of poor instruction of scientific research in undergraduate medical curricula, tendency of prospective healthcare providers to carry out pertinently applicable evidence-based medicine will be impossible. This will consequently impact negatively in practicing relevant evidence-based medicine and accordingly endanger/jeopardize the best possible conduct of clinical practice. Furthermore, and most importantly, it must be pointed out that the practice of evidence-based medicine guided by adequate research competencies is one of the keys to generate proficient, and most significantly, ‘safe doctors’.

As ‘practice makes perfect’, medical students should be introduced to scientific research as early as possible in undergraduate medical education as well as engaged in as many various curricular/extracurricular undergraduate research activities. This is because in the settings of early introduction to undergraduate research, knowledge of scientific research and familiarity with basic research skills, medical students will be increasingly self-assured to continue further research activities and gradually solidify and build up their scientific research competencies over time.

It must be noted that no prospective medical student must be obliged to do research, as this may promote a tendency for ‘bad research’ negatively influencing the academic and research institutes. However, basics of scientific research to largely comprehend research studies, critically appraise findings and wisely deduce conclusions should be offered in undergraduate medical curricula. Research-related ethical matters should be also considered in undergraduate medical curricula.

It must be recognized that educators of undergraduate medical curricula hold the chief responsibility in nurturing scientific research competencies in medical students. Such nurturing of scientific research competencies should start early in undergraduate medical education and span throughout the pre-clinical and clinical years of undergraduate medical education. This is because medical students, with the passage of time, tend to forget the taught research competencies if they are not somehow revisited, revised or utilized appropriately. Failure to develop positive attitudes of scientific research will influence negatively on the curiosity of students in considering further research activities or on commencing research-focused careers. Moreover, as proper execution of evidence-based medicine is largely based on solid acquisition of scientific research competencies, failure to develop such competencies will most likely weaken the proper practice of medicine.

Teaching scientific research in undergraduate medical curricula (possibly as an individual-based accredited course) is highly advised and has been coupled with positive attitudes towards research, advancement of research competencies, and smooth facilitation to engagement in future research undertakings (8). Basic knowledge about information systems, epidemiology, biostatistics, current challenges in clinical medicine, and codes of ethics implicated in the best conduct of clinical research with human subjects should be provided. Furthermore, raising awareness of matters pertaining to potential research-related conflicts of interest and ethical misconducts should be also adequately presented.

Many medical students fail to appreciate the important concept of ‘translational research’ and its key players ‘physician–scientists’. Translational research integrates advances in biomedical and clinical sciences, and translates these findings from the ‘bench-to-bedside’ – that is, from laboratory benches and clinical trials to pertinently relevant and beneficial patient care applications (17). Physicians–scientists, with one foot in clinical practice, and one foot in biomedical/clinical research, are distinctively oriented to promote ‘bench-to-bedside’ scientific research ventures, speed up groundbreaking innovations, expand evidence-based medicine and radically advance healthcare diagnostics and therapeutics.

Taking into account the importance of translational research and the absolute need to produce physician–scientist generations, there has been a rapidly evolving emphasis towards incorporating a ‘translational research’ instructional component into medical curricula. The aim of such emphasis is to sufficiently introduce prospective medical doctors to the fundamental scientific and ethical standards of translational research, including the means to how such research is properly conducted, critically evaluated, appropriately explained to patients, and effectively/productively applied to patient care.

In conclusion, despite the importance and benefits of undergraduate research, attempts of medical schools to encourage undergraduates to take part in formal research training during undergraduate medical education remain unsatisfactory. It is highly recommended to integrate formal research training into undergraduate medical curricula and to provide a diversity of high-quality well-mentored undergraduate research opportunities. Instruction of research knowledge and skills in undergraduate medical curricula is absolutely indispensable to the proper practice of evidence-based medicine and effective carrying out of clinical practice. Furthermore, it is very important to drive students to develop positive attitudes towards undergraduate research and to resolve any barrier that may hinder medical students from productive engagement in undergraduate research activities.
